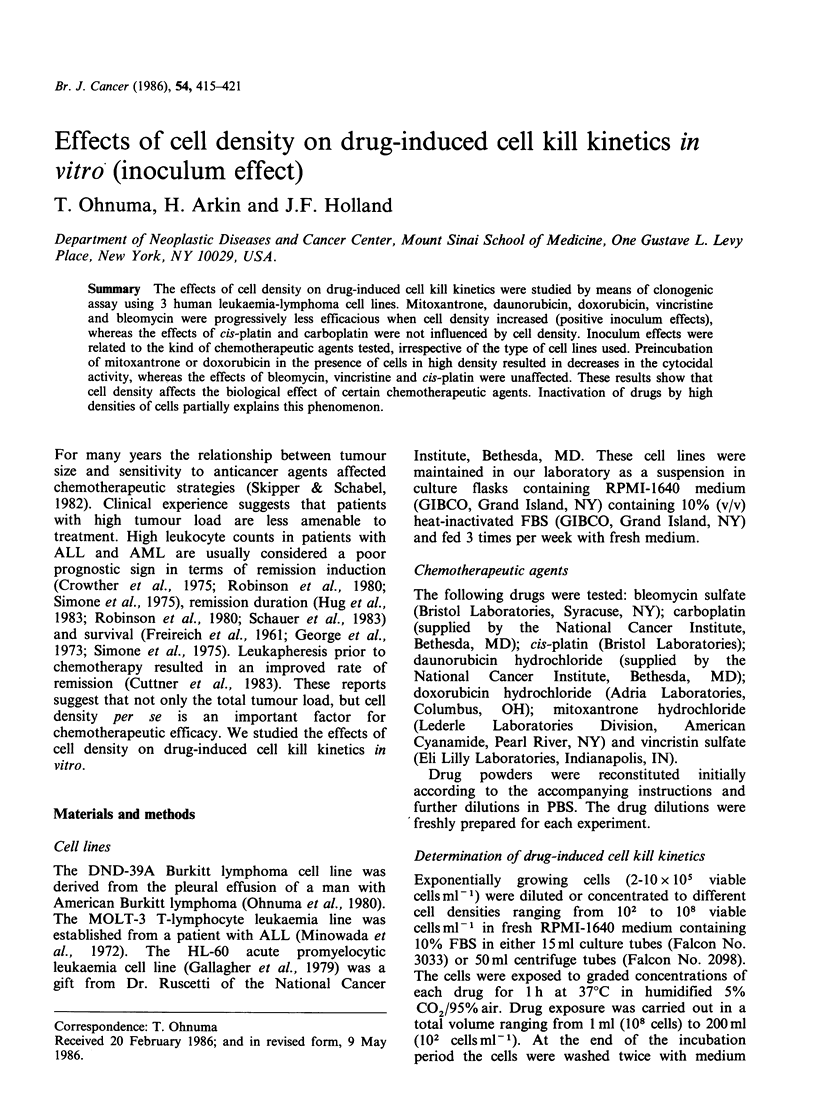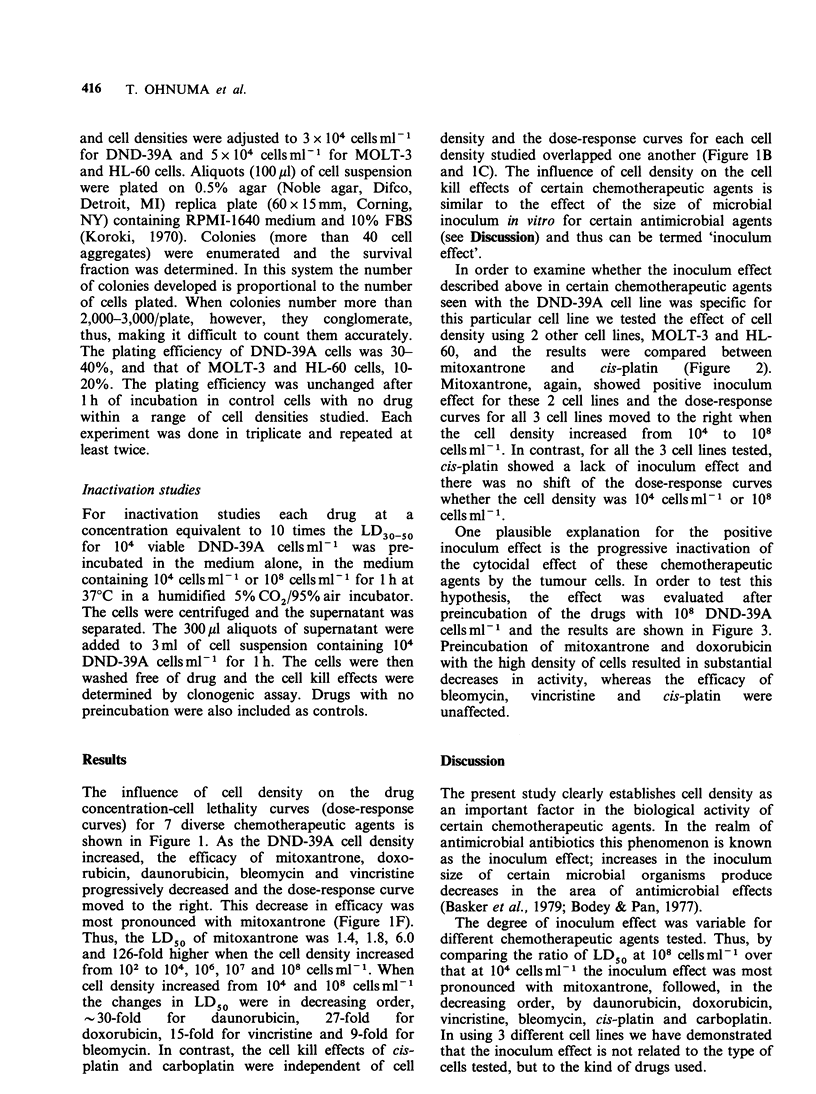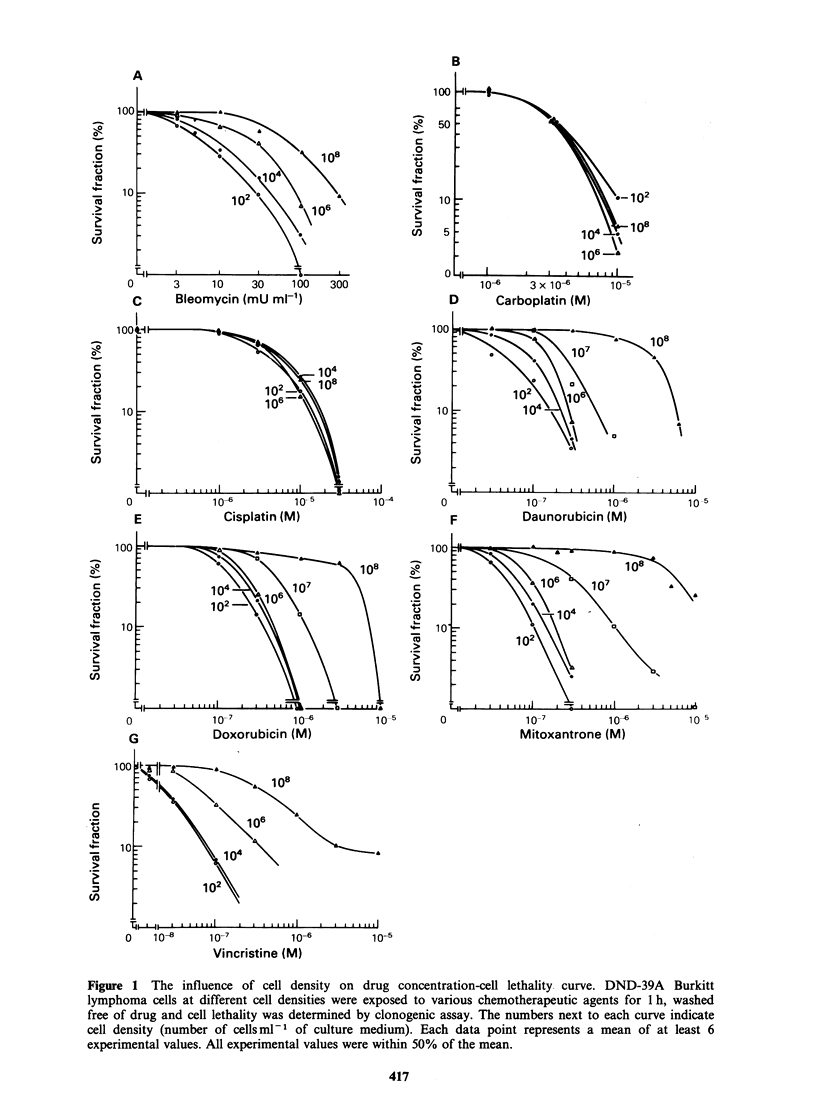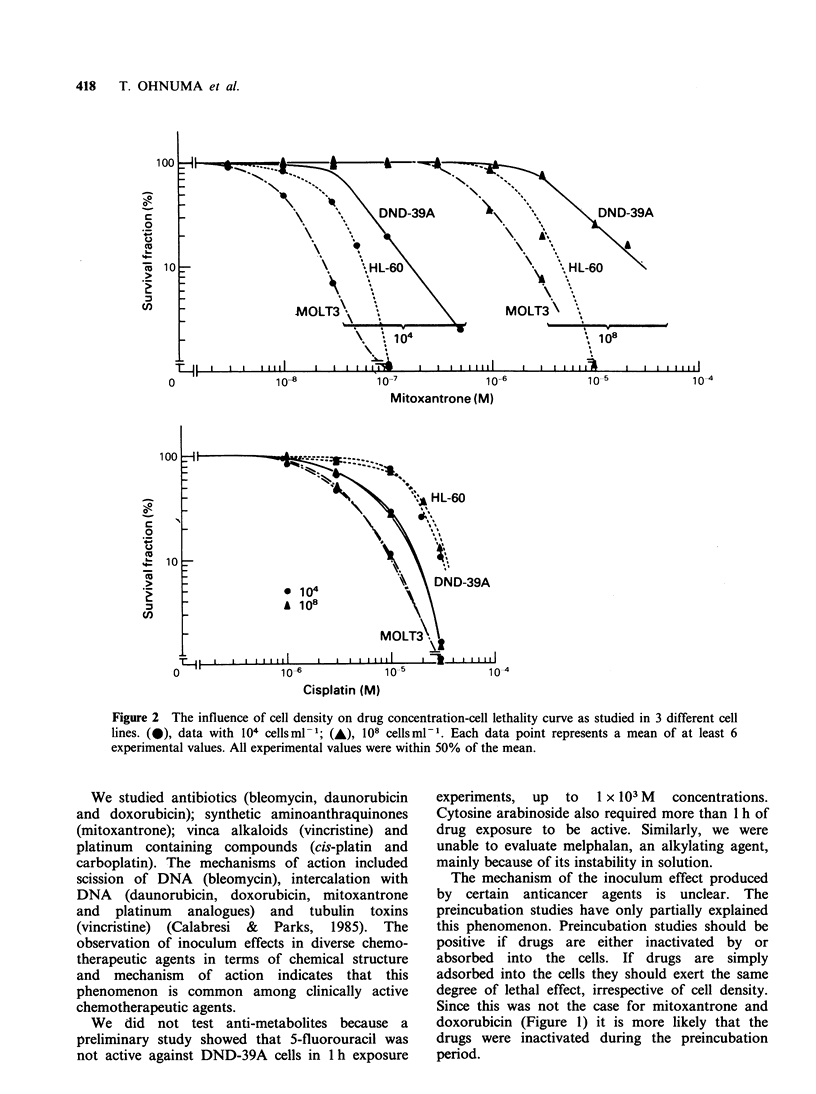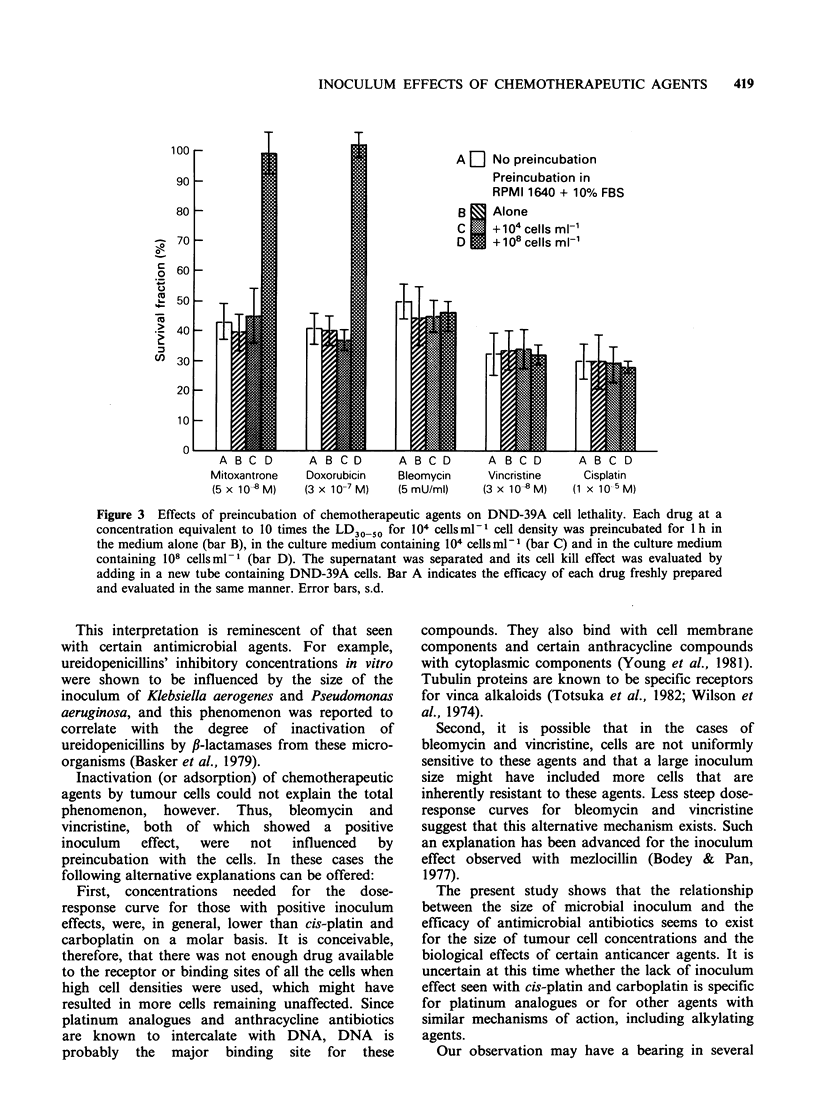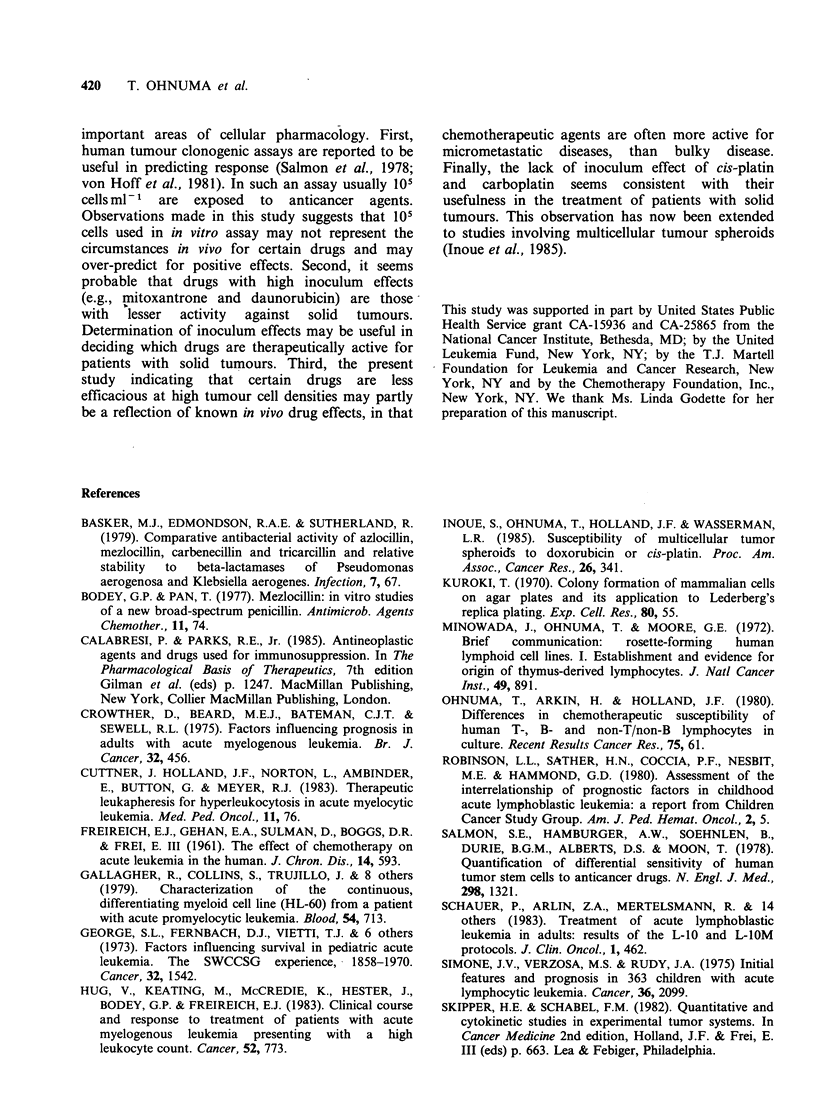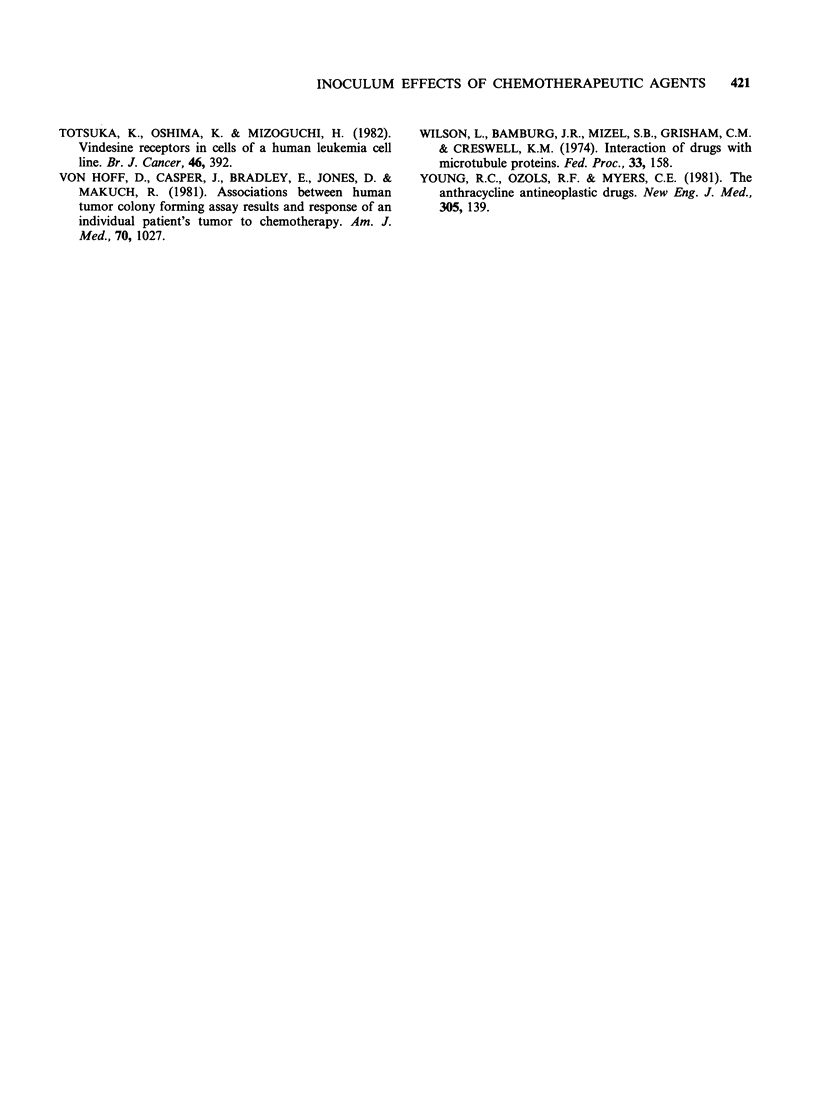# Effects of cell density on drug-induced cell kill kinetics in vitro (inoculum effect).

**DOI:** 10.1038/bjc.1986.192

**Published:** 1986-09

**Authors:** T. Ohnuma, H. Arkin, J. F. Holland

## Abstract

The effects of cell density on drug-induced cell kill kinetics were studied by means of clonogenic assay using 3 human leukaemia-lymphoma cell lines. Mitoxantrone, daunorubicin, doxorubicin, vincristine and bleomycin were progressively less efficacious when cell density increased (positive inoculum effects), whereas the effects of cis-platin and carboplatin were not influenced by cell density. Inoculum effects were related to the kind of chemotherapeutic agents tested, irrespective of the type of cell lines used. Preincubation of mitoxantrone or doxorubicin in the presence of cells in high density resulted in decreases in the cytocidal activity, whereas the effects of bleomycin, vincristine and cis-platin were unaffected. These results show that cell density affects the biological effect of certain chemotherapeutic agents. Inactivation of drugs by high densities of cells partially explains this phenomenon.


*Br. J. Cancer* (1986), **54**, 415–421

### Effects of cell density on drug-induced cell kill kinetics *in vitro* (inoculum effect)

T. Ohnuma, H. Arkin and J.F. Holland

*Department of Neoplastic Diseases and Cancer Center, Mount Sinai School of Medicine, One Gustave L. Levy Place, New York, NY 10029, USA.*

**Summary** The effects of cell density on drug-induced cell kill kinetics were studied by means of clonogenic assay using 3 human leukaemia-lymphoma cell lines. Mitoxantrone, daunorubicin, doxorubicin, vincristine and bleomycin were progressively less efficacious when cell density increased (positive inoculum effects), whereas the effects of *cis*-platin and carboplatin were not influenced by cell density. Inoculum effects were related to the kind of chemotherapeutic agents tested, irrespective of the type of cell lines used. Preincubation of mitoxantrone or doxorubicin in the presence of cells in high density resulted in decreases in the cytocidal activity, whereas the effects of bleomycin, vincristine and *cis*-platin were unaffected. These results show that cell density affects the biological effect of certain chemotherapeutic agents. Inactivation of drugs by high densities of cells partially explains this phenomenon.

For many years the relationship between tumour size and sensitivity to anticancer agents affected chemotherapeutic strategies (Skipper & Schabel, 1982). Clinical experience suggests that patients with high tumour load are less amenable to treatment. High leukocyte counts in patients with ALL and AML are usually considered a poor prognostic sign in terms of remission induction (Crowther *et al.*, 1975; Robinson *et al.*, 1980; Simone *et al.*, 1975), remission duration (Hug *et al.*, 1983; Robinson *et al.*, 1980; Schauer *et al.*, 1983) and survival (Freireich *et al.*, 1961; George *et al.*, 1973; Simone *et al.*, 1975). Leukapheresis prior to chemotherapy resulted in an improved rate of remission (Cuttner *et al.*, 1983). These reports suggest that not only the total tumour load, but cell density *per se* is an important factor for chemotherapeutic efficacy. We studied the effects of cell density on drug-induced cell kill kinetics *in vitro*.

### Materials and methods

### *Cell lines*

The DND-39A Burkitt lymphoma cell line was derived from the pleural effusion of a man with American Burkitt lymphoma (Ohnuma *et al.*, 1980). The MOLT-3 T-lymphocyte leukaemia line was established from a patient with ALL (Minowada *et al.*, 1972). The HL-60 acute promyelocytic leukaemia cell line (Gallagher *et al.*, 1979) was a gift from Dr. Ruscetti of the National Cancer Institute, Bethesda, MD. These cell lines were maintained in our laboratory as a suspension in culture flasks containing RPMI-1640 medium (GIBCO, Grand Island, NY) containing 10% (v/v) heat-inactivated FBS (GIBCO, Grand Island, NY) and fed 3 times per week with fresh medium.

### *Chemotherapeutic agents*

The following drugs were tested: bleomycin sulfate (Bristol Laboratories, Syracuse, NY); carboplatin (supplied by the National Cancer Institute, Bethesda, MD); *cis*-platin (Bristol Laboratories); daunorubicin hydrochloride (supplied by the National Cancer Institute, Bethesda, MD); doxorubicin hydrochloride (Adria Laboratories, Columbus, OH); mitoxantrone hydrochloride (Lederle Laboratories Division, American Cyanamide, Pearl River, NY) and vincristin sulfate (Eli Lilly Laboratories, Indianapolis, IN).

Drug powders were reconstituted initially according to the accompanying instructions and further dilutions in PBS. The drug dilutions were freshly prepared for each experiment.

### *Determination of drug-induced cell kill kinetics*

Exponentially growing cells ($2\text{-}10 \times 10^5$ viable cells ml$^{-1}$) were diluted or concentrated to different cell densities ranging from $10^2$ to $10^8$ viable cells ml$^{-1}$ in fresh RPMI-1640 medium containing 10% FBS in either 15 ml culture tubes (Falcon No. 3033) or 50 ml centrifuge tubes (Falcon No. 2098). The cells were exposed to graded concentrations of each drug for 1 h at 37°C in humidified 5% $CO_2$/95% air. Drug exposure was carried out in a total volume ranging from 1 ml ($10^8$ cells) to 200 ml ($10^2$ cells ml$^{-1}$). At the end of the incubation period the cells were washed twice with medium

Correspondence: T. Ohnuma
Received 20 February 1986; and in revised form, 9 May 1986.

and cell densities were adjusted to $3 \times 10^4$ cells ml$^{-1}$ for DND-39A and $5 \times 10^4$ cells ml$^{-1}$ for MOLT-3 and HL-60 cells. Aliquots (100 $\mu$l) of cell suspension were plated on 0.5% agar (Noble agar, Difco, Detroit, MI) replica plate (60 × 15 mm, Corning, NY) containing RPMI-1640 medium and 10% FBS (Koroki, 1970). Colonies (more than 40 cell aggregates) were enumerated and the survival fraction was determined. In this system the number of colonies developed is proportional to the number of cells plated. When colonies number more than 2,000–3,000/plate, however, they conglomerate, thus, making it difficult to count them accurately. The plating efficiency of DND-39A cells was 30–40%, and that of MOLT-3 and HL-60 cells, 10-20%. The plating efficiency was unchanged after 1 h of incubation in control cells with no drug within a range of cell densities studied. Each experiment was done in triplicate and repeated at least twice.

*Inactivation studies*

For inactivation studies each drug at a concentration equivalent to 10 times the $LD_{30-50}$ for $10^4$ viable DND-39A cells ml$^{-1}$ was pre-incubated in the medium alone, in the medium containing $10^4$ cells ml$^{-1}$ or $10^8$ cells ml$^{-1}$ for 1 h at 37°C in a humidified 5% $CO_2$/95% air incubator. The cells were centrifuged and the supernatant was separated. The 300 $\mu$l aliquots of supernatant were added to 3 ml of cell suspension containing $10^4$ DND-39A cells ml$^{-1}$ for 1 h. The cells were then washed free of drug and the cell kill effects were determined by clonogenic assay. Drugs with no preincubation were also included as controls.

### Results

The influence of cell density on the drug concentration-cell lethality curves (dose-response curves) for 7 diverse chemotherapeutic agents is shown in Figure 1. As the DND-39A cell density increased, the efficacy of mitoxantrone, doxorubicin, daunorubicin, bleomycin and vincristine progressively decreased and the dose-response curve moved to the right. This decrease in efficacy was most pronounced with mitoxantrone (Figure 1F). Thus, the $LD_{50}$ of mitoxantrone was 1.4, 1.8, 6.0 and 126-fold higher when the cell density increased from $10^2$ to $10^4$, $10^6$, $10^7$ and $10^8$ cells ml$^{-1}$. When cell density increased from $10^4$ and $10^8$ cells ml$^{-1}$ the changes in $LD_{50}$ were in decreasing order, ~30-fold for daunorubicin, 27-fold for doxorubicin, 15-fold for vincristine and 9-fold for bleomycin. In contrast, the cell kill effects of *cis*-platin and carboplatin were independent of cell density and the dose-response curves for each cell density studied overlapped one another (Figure 1B and 1C). The influence of cell density on the cell kill effects of certain chemotherapeutic agents is similar to the effect of the size of microbial inoculum *in vitro* for certain antimicrobial agents (see **Discussion**) and thus can be termed 'inoculum effect'.

In order to examine whether the inoculum effect described above in certain chemotherapeutic agents seen with the DND-39A cell line was specific for this particular cell line we tested the effect of cell density using 2 other cell lines, MOLT-3 and HL-60, and the results were compared between mitoxantrone and *cis*-platin (Figure 2). Mitoxantrone, again, showed positive inoculum effect for these 2 cell lines and the dose-response curves for all 3 cell lines moved to the right when the cell density increased from $10^4$ to $10^8$ cells ml$^{-1}$. In contrast, for all the 3 cell lines tested, *cis*-platin showed a lack of inoculum effect and there was no shift of the dose-response curves whether the cell density was $10^4$ cells ml$^{-1}$ or $10^8$ cells ml$^{-1}$.

One plausible explanation for the positive inoculum effect is the progressive inactivation of the cytocidal effect of these chemotherapeutic agents by the tumour cells. In order to test this hypothesis, the effect was evaluated after preincubation of the drugs with $10^8$ DND-39A cells ml$^{-1}$ and the results are shown in Figure 3. Preincubation of mitoxantrone and doxorubicin with the high density of cells resulted in substantial decreases in activity, whereas the efficacy of bleomycin, vincristine and *cis*-platin were unaffected.

### Discussion

The present study clearly establishes cell density as an important factor in the biological activity of certain chemotherapeutic agents. In the realm of antimicrobial antibiotics this phenomenon is known as the inoculum effect; increases in the inoculum size of certain microbial organisms produce decreases in the area of antimicrobial effects (Basker *et al.*, 1979; Bodey & Pan, 1977).

The degree of inoculum effect was variable for different chemotherapeutic agents tested. Thus, by comparing the ratio of $LD_{50}$ at $10^8$ cells ml$^{-1}$ over that at $10^4$ cells ml$^{-1}$ the inoculum effect was most pronounced with mitoxantrone, followed, in the decreasing order, by daunorubicin, doxorubicin, vincristine, bleomycin, *cis*-platin and carboplatin. In using 3 different cell lines we have demonstrated that the inoculum effect is not related to the type of cells tested, but to the kind of drugs used.

**Figure 1** The influence of cell density on drug concentration-cell lethality curve. DND-39A Burkitt lymphoma cells at different cell densities were exposed to various chemotherapeutic agents for 1 h, washed free of drug and cell lethality was determined by clonogenic assay. The numbers next to each curve indicate cell density (number of cells $ml^{-1}$ of culture medium). Each data point represents a mean of at least 6 experimental values. All experimental values were within 50% of the mean.

**Figure 2** The influence of cell density on drug concentration-cell lethality curve as studied in 3 different cell lines. (●), data with $10^4$ cells $ml^{-1}$; (▲), $10^8$ cells $ml^{-1}$. Each data point represents a mean of at least 6 experimental values. All experimental values were within 50% of the mean.

We studied antibiotics (bleomycin, daunorubicin and doxorubicin); synthetic aminoanthraquinones (mitoxantrone); vinca alkaloids (vincristine) and platinum containing compounds (*cis*-platin and carboplatin). The mechanisms of action included scission of DNA (bleomycin), intercalation with DNA (daunorubicin, doxorubicin, mitoxantrone and platinum analogues) and tubulin toxins (vincristine) (Calabresi & Parks, 1985). The observation of inoculum effects in diverse chemotherapeutic agents in terms of chemical structure and mechanism of action indicates that this phenomenon is common among clinically active chemotherapeutic agents.

We did not test anti-metabolites because a preliminary study showed that 5-fluorouracil was not active against DND-39A cells in 1 h exposure experiments, up to $1 \times 10^3$ M concentrations. Cytosine arabinoside also required more than 1 h of drug exposure to be active. Similarly, we were unable to evaluate melphalan, an alkylating agent, mainly because of its instability in solution.

The mechanism of the inoculum effect produced by certain anticancer agents is unclear. The preincubation studies have only partially explained this phenomenon. Preincubation studies should be positive if drugs are either inactivated by or absorbed into the cells. If drugs are simply adsorbed into the cells they should exert the same degree of lethal effect, irrespective of cell density. Since this was not the case for mitoxantrone and doxorubicin (Figure 1) it is more likely that the drugs were inactivated during the preincubation period.

**Figure 3** Effects of preincubation of chemotherapeutic agents on DND-39A cell lethality. Each drug at a concentration equivalent to 10 times the $LD_{30-50}$ for $10^4$ cells $ml^{-1}$ cell density was preincubated for 1 h in the medium alone (bar B), in the culture medium containing $10^4$ cells $ml^{-1}$ (bar C) and in the culture medium containing $10^8$ cells $ml^{-1}$ (bar D). The supernatant was separated and its cell kill effect was evaluated by adding in a new tube containing DND-39A cells. Bar A indicates the efficacy of each drug freshly prepared and evaluated in the same manner. Error bars, s.d.

This interpretation is reminescent of that seen with certain antimicrobial agents. For example, ureidopenicillins' inhibitory concentrations *in vitro* were shown to be influenced by the size of the inoculum of *Klebsiella aerogenes* and *Pseudomonas aeruginosa*, and this phenomenon was reported to correlate with the degree of inactivation of ureidopenicillins by β-lactamases from these micro-organisms (Basker *et al.*, 1979).

Inactivation (or adsorption) of chemotherapeutic agents by tumour cells could not explain the total phenomenon, however. Thus, bleomycin and vincristine, both of which showed a positive inoculum effect, were not influenced by preincubation with the cells. In these cases the following alternative explanations can be offered:

First, concentrations needed for the dose-response curve for those with positive inoculum effects, were, in general, lower than *cis*-platin and carboplatin on a molar basis. It is conceivable, therefore, that there was not enough drug available to the receptor or binding sites of all the cells when high cell densities were used, which might have resulted in more cells remaining unaffected. Since platinum analogues and anthracycline antibiotics are known to intercalate with DNA, DNA is probably the major binding site for these compounds. They also bind with cell membrane components and certain anthracycline compounds with cytoplasmic components (Young *et al.*, 1981). Tubulin proteins are known to be specific receptors for vinca alkaloids (Totsuka *et al.*, 1982; Wilson *et al.*, 1974).

Second, it is possible that in the cases of bleomycin and vincristine, cells are not uniformly sensitive to these agents and that a large inoculum size might have included more cells that are inherently resistant to these agents. Less steep dose-response curves for bleomycin and vincristine suggest that this alternative mechanism exists. Such an explanation has been advanced for the inoculum effect observed with mezlocillin (Bodey & Pan, 1977).

The present study shows that the relationship between the size of microbial inoculum and the efficacy of antimicrobial antibiotics seems to exist for the size of tumour cell concentrations and the biological effects of certain anticancer agents. It is uncertain at this time whether the lack of inoculum effect seen with *cis*-platin and carboplatin is specific for platinum analogues or for other agents with similar mechanisms of action, including alkylating agents.

Our observation may have a bearing in several important areas of cellular pharmacology. First, human tumour clonogenic assays are reported to be useful in predicting response (Salmon *et al.*, 1978; von Hoff *et al.*, 1981). In such an assay usually $10^5$ cells $ml^{-1}$ are exposed to anticancer agents. Observations made in this study suggests that $10^5$ cells used in *in vitro* assay may not represent the circumstances *in vivo* for certain drugs and may over-predict for positive effects. Second, it seems probable that drugs with high inoculum effects (e.g., mitoxantrone and daunorubicin) are those with lesser activity against solid tumours. Determination of inoculum effects may be useful in deciding which drugs are therapeutically active for patients with solid tumours. Third, the present study indicating that certain drugs are less efficacious at high tumour cell densities may partly be a reflection of known *in vivo* drug effects, in that chemotherapeutic agents are often more active for micrometastatic diseases, than bulky disease. Finally, the lack of inoculum effect of *cis*-platin and carboplatin seems consistent with their usefulness in the treatment of patients with solid tumours. This observation has now been extended to studies involving multicellular tumour spheroids (Inoue *et al.*, 1985).

This study was supported in part by United States Public Health Service grant CA-15936 and CA-25865 from the National Cancer Institute, Bethesda, MD; by the United Leukemia Fund, New York, NY; by the T.J. Martell Foundation for Leukemia and Cancer Research, New York, NY and by the Chemotherapy Foundation, Inc., New York, NY. We thank Ms. Linda Godette for her preparation of this manuscript.

### References

BASKER, M.J., EDMONDSON, R.A.E. & SUTHERLAND, R. (1979). Comparative antibacterial activity of azlocillin, mezlocillin, carbenecillin and tricarcillin and relative stability to beta-lactamases of Pseudomonas aerogenosa and Klebsiella aerogenes. *Infection*, **7**, 67.

BODEY, G.P. & PAN, T. (1977). Mezlocillin: in vitro studies of a new broad-spectrum penicillin. *Antimicrob. Agents Chemother.*, **11**, 74.

CALABRESI, P. & PARKS, R.E., Jr. (1985). Antineoplastic agents and drugs used for immunosuppression. In *The Pharmacological Basis of Therapeutics*, 7th edition Gilman *et al.* (eds) p. 1247. MacMillan Publishing, New York, Collier MacMillan Publishing, London.

CROWTHER, D., BEARD, M.E.J., BATEMAN, C.J.T. & SEWELL, R.L. (1975). Factors influencing prognosis in adults with acute myelogenous leukemia. *Br. J. Cancer*, **32**, 456.

CUTTNER, J. HOLLAND, J.F., NORTON, L., AMBINDER, E., BUTTON, G. & MEYER, R.J. (1983). Therapeutic leukapheresis for hyperleukocytosis in acute myelocytic leukemia. *Med. Ped. Oncol.*, **11**, 76.

FREIREICH, E.J., GEHAN, E.A., SULMAN, D., BOGGS, D.R. & FREI, E. III (1961). The effect of chemotherapy on acute leukemia in the human. *J. Chron. Dis.*, **14**, 593.

GALLAGHER, R., COLLINS, S., TRUJILLO, J. & 8 others (1979). Characterization of the continuous, differentiating myeloid cell line (HL-60) from a patient with acute promyelocytic leukemia. *Blood*, **54**, 713.

GEORGE, S.L., FERNBACH, D.J., VIETTI, T.J. & 6 others (1973). Factors influencing survival in pediatric acute leukemia. The SWCCSG experience, 1858–1970. *Cancer*, **32**, 1542.

HUG, V., KEATING, M., McCREDIE, K., HESTER, J., BODEY, G.P. & FREIREICH, E.J. (1983). Clinical course and response to treatment of patients with acute myelogenous leukemia presenting with a high leukocyte count. *Cancer*, **52**, 773.

INOUE, S., OHNUMA, T., HOLLAND, J.F. & WASSERMAN, L.R. (1985). Susceptibility of multicellular tumor spheroids to doxorubicin or *cis*-platin. *Proc. Am. Assoc., Cancer Res.*, **26**, 341.

KUROKI, T. (1970). Colony formation of mammalian cells on agar plates and its application to Lederberg's replica plating. *Exp. Cell. Res.*, **80**, 55.

MINOWADA, J., OHNUMA, T. & MOORE, G.E. (1972). Brief communication: rosette-forming human lymphoid cell lines. I. Establishment and evidence for origin of thymus-derived lymphocytes. *J. Natl Cancer Inst.*, **49**, 891.

OHNUMA, T., ARKIN, H. & HOLLAND, J.F. (1980). Differences in chemotherapeutic susceptibility of human T-, B- and non-T/non-B lymphocytes in culture. *Recent Results Cancer Res.*, **75**, 61.

ROBINSON, L.L., SATHER, H.N., COCCIA, P.F., NESBIT, M.E. & HAMMOND, G.D. (1980). Assessment of the interrelationship of prognostic factors in childhood acute lymphoblastic leukemia: a report from Children Cancer Study Group. *Am. J. Ped. Hemat. Oncol.*, **2**, 5.

SALMON, S.E., HAMBURGER, A.W., SOEHNLEN, B., DURIE, B.G.M., ALBERTS, D.S. & MOON, T. (1978). Quantification of differential sensitivity of human tumor stem cells to anticancer drugs. *N. Engl. J. Med.*, **298**, 1321.

SCHAUER, P., ARLIN, Z.A., MERTELSMANN, R. & 14 others (1983). Treatment of acute lymphoblastic leukemia in adults: results of the L-10 and L-10M protocols. *J. Clin. Oncol.*, **1**, 462.

SIMONE, J.V., VERZOSA, M.S. & RUDY, J.A. (1975) Initial features and prognosis in 363 children with acute lymphocytic leukemia. *Cancer*, **36**, 2099.

SKIPPER, H.E. & SCHABEL, F.M. (1982). Quantitative and cytokinetic studies in experimental tumor systems. In *Cancer Medicine* 2nd edition, Holland, J.F. & Frei, E. III (eds) p. 663. Lea & Febiger, Philadelphia.

TOTSUKA, K., OSHIMA, K. & MIZOGUCHI, H. (1982). Vindesine receptors in cells of a human leukemia cell line. *Br. J. Cancer*, **46**, 392.

VON HOFF, D., CASPER, J., BRADLEY, E., JONES, D. & MAKUCH, R. (1981). Associations between human tumor colony forming assay results and response of an individual patient's tumor to chemotherapy. *Am. J. Med.*, **70**, 1027.

WILSON, L., BAMBURG, J.R., MIZEL, S.B., GRISHAM, C.M. & CRESWELL, K.M. (1974). Interaction of drugs with microtubule proteins. *Fed. Proc.*, **33**, 158.

YOUNG, R.C., OZOLS, R.F. & MYERS, C.E. (1981). The anthracycline antineoplastic drugs. *New Eng. J. Med.*, **305**, 139.